# Identification and mapping of yield and yield related QTLs from an Indian accession of *Oryza rufipogon*

**DOI:** 10.1186/1471-2156-6-33

**Published:** 2005-06-13

**Authors:** Pradeep Reddy Marri, Sarla N, Laxminarayana V Reddy, EA Siddiq

**Affiliations:** 1 National Professor Project, Directorate of Rice Research, Rajendranagar, Hyderabad 500 030, India

## Abstract

**Background:**

Cultivated rice (*Oryza sativa *L.) is endowed with a rich genetic variability. In spite of such a great diversity, the modern rice cultivars have narrow genetic base for most of the agronomically important traits. To sustain the demand of an ever increasing population, new avenues have to be explored to increase the yield of rice. Wild progenitor species present potential donor sources for complex traits such as yield and would help to realize the dream of sustained food security.

**Results:**

Advanced backcross method was used to introgress and map new quantitative trait loci (QTLs) relating to yield and its components from an Indian accession of Oryza *rufipogon*. An interspecific BC_2_ testcross progeny (IR58025A/*O. rufipogon*//IR580325B///IR58025B////KMR3) was evaluated for 13 agronomic traits pertaining to yield and its components. Transgressive segregants were obtained for all the traits. Thirty nine QTLs were identified using interval mapping and composite interval mapping. In spite of it's inferiority for most of the traits studied, *O. rufipogon *alleles contributed positively to 74% of the QTLs. Thirty QTLs had corresponding occurrences with the QTLs reported earlier, indicating that these QTLs are stable across genetic backgrounds. Nine QTLs are novel and reported for the first time.

**Conclusion:**

The study confirms that the progenitor species constitute a prominent source of still unfolded variability for traits of complex inheritance like yield. With the availability of the complete genome sequence of rice and the developments in the field of genomics, it is now possible to identify the genes underlying the QTLs. The identification of the genes constituting QTLs would help us to understand the molecular mechanisms behind the action of QTLs.

## Background

The modern day cultivars of rice, in spite of all their high yielding potential and other desirable features are handicapped with narrow genetic base for most of the agronomically important traits including the dwarf habit, which is the major yield enhancing trait. Recent study of high yielding Indian rice varieties for their ancestry revealed that hardly 5 to 6 accessions accounted for more than 90% of their genetic constitution, confirming that the cultivar gene pool being depended on now for improvement represent hardly 15% of the total genetic variability available in rice germplasm (E A Siddiq, personal communication). Rice is endowed with very rich genetic diversity. Wild/weedy species along with very large number of primitive cultivars and landraces constitute an important reservoir of useful genes. The size of additional variability they can provide would be of great value to the ongoing crop improvement endeavor. Large genetic variability still remains untapped in the wild relatives and primitive cultivars of rice [1]. Considering the large hidden variability and very rare and agronomically important genes they possibly possess, utilization of the wild species is critical to future crop improvement [2]. Utilization of these exotic species as donors in interspecific crosses is one of the strategies to harness their hidden potential and broaden the genetic diversity of the existing gene pool. Over the last decade, wild species in rice have been successfully utilized for introgression of diverse traits such cytoplasmic male sterility (cms) [3-6], abiotic and biotic stress [7-11], yield and its components [12-18] and grain quality [19-21] into the cultivars. A great deal of work in the recent past, on the wild species of rice, concentrated on the utilization of these species for quantitative traits such as yield and its components long with grain quality. In the first ever report on the use of wild species for introgression of quantitative characters, two yield QTLs, *yld1.1 and yld 2.1*, each of which is capable of increasing yield by about 18% have been identified in a Malaysian accession of *O. rufipogon *[12,13]. This was a precursor to many studies resulting in the identification of numerous QTLs pertaining to yield and grain quality [12-21]. Keeping in view the unlimited potential of wild/weedy species of rice for yield genes as evident from the foregoing research, the present study reports the identification and mapping of molecular marker-associated yield QTLs in an Indian accession of *O. rufipogon *(IC 22015). An interspecific testcross population, derived using an advanced backcross QTL strategy (AB-QTL) [22], between *O. rufipogon *and IR 58025A, a widely used cms line in India, was used to map QTLs related to yield and it's components. The AB-QTL method has been successfully employed earlier in tomato and rice to transfer positive alleles from phenotypically inferior wild and weedy species into elite cultivars [23-25,14-19]. In addition to identifying potential novel QTLs for yield and it's components, the results from the current study will provide additional data for comparison with QTLs that are previously documented in rice. Comparisons across different genetic backgrounds will provide information about the conservation of QTLs and help us to understand the interactions of QTL alleles across multiple backgrounds and environments.

## Results

### Trait analysis and field performance

**Figure 1:**
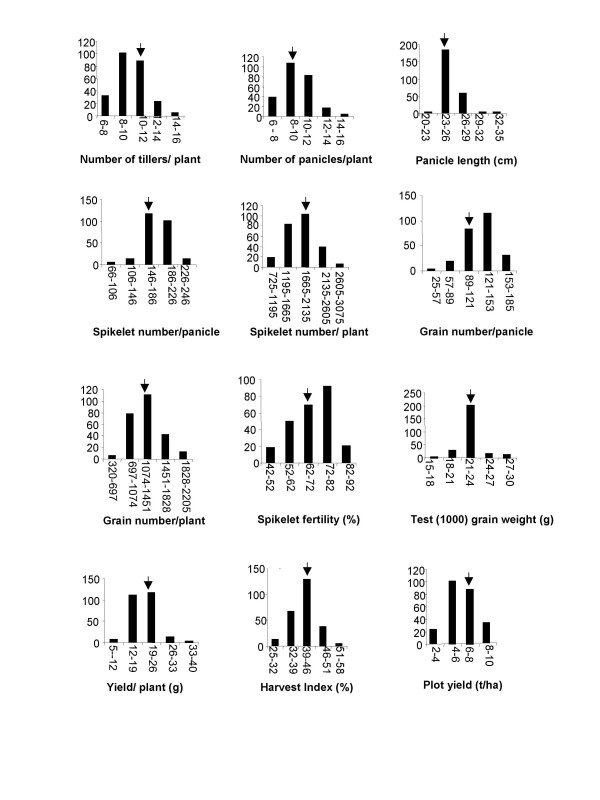
Frequency distribution of the 251 testcross families for yield and its components. Arrow indicates the value of the hybrid check, KRH2. Y-axis: Number of individuals.

The phenotypic analysis of the 251 testcross families showed that the frequency Distribution of all traits approximately fit normal distribution (Figure 1). As expected in an interpsecific cross, character wise frequency distribution of testcross families showed transgressive segregants for all the traits. For a depiction of variation in tiller number and panicle length in the testcross families, see additional file 1. The average grain yield of the testcross families was 6.08 t/ha, with the range varying from 3.90 to 9.45 t/ha, while yield per plant ranged from 7.5 to 36.0 g with an average of 19.5 g. Thirteen testcross families outperformed the hybrid check, KRH2, by more than 20% for plot yield and as many as 39 families showed more than 20% increase in yield per plant as compared to KRH2 (Table 1). Of the 251 testcross families studied in all, 75 showed at least 20% increase over KRH2 for three or more yield components.

**Table 1:** Mean phenotypic traits for 13 yield components across 251 testcross families as compared to IR 58025A, IC22015 (wild) and KRH2

Trait	**IR 58025A^a^**	**IC 22015^b^**	**KRH2^c^**	**Range in Testcross families**	**No. of families showing >20% increase over KRH2**
Plant height (cm)	80	119	118	93 – 177	26
Number of tillers	9	32	11.2	7 – 16	18
Number of panicles	7	28	10	6 – 14	20
Panicle length (cm)	24	29	23.5	20.5 – 34.5	1
Spikelet number/panicle	175	150	167	67 – 265	88
Spikelet number/plant	1350	3500	1880	737 – 3074	74
Grain number/panicle	152*	35	117	30 – 185	101
Grain number/plant	1064*	700	1187	322 – 2310	63
Spikelet fertility (%)	0	15–20	68	42 – 91	42
1000 grain weight (g)	20	11.5	22.5	17.5 – 31.3	1
Yield / plant (g)	16*	9	19	7.5 – 36	39
Harvest index	32*	-	45	27 – 56	7
Yield (t/ha)	4.2	-	7	3.9 – 9.45	13

cm: Centimeters, g: Grams, t/ha: Tonnes / hectare

a – *O. sativa *parent, b – *O. rufipogon *parent, c- hybrid check

* for the traits where IR 58025A has no corresponding value, the value from IR 58025B, an isogenic line of IR 58025A is used.

### Trait correlations

The trait correlations confirmed to the expected results. Significant positive correlations (P < 0.01) included SPY (Single plant yield) × SNP (Spikelet number per plant) (0.552), SPY × GNP (Grain number per plant) (0.581) and HI (Harvest index) × SPY (0.539) whereas, the significant negatively correlated traits included PH (Plant height) × SF (Spikelet fertility) (-0.362), GW (Grain weight) × NT (Number of tillers) (-0.255), GW × NP (Number of panicles) (-0.284) and HI × PH (-0.298). Interestingly GW had no significant effect on PY (Plot yield), but showed negative correlation with NP. For detailed character pair correlations among the traits see additional file 2.

### Marker polymorphism

Two hundred and ten microsatellite markers were used to screen the parents for identifying polymorphic markers. Eighty markers (38%) detected polymorphism. The polymorphism is lower compared to earlier studies involving *O. rufipogon*, where the polymorphism ranged from 60–90% [13,15,17]. Polymorphism is a measure of genetic diversity and varies with the parental combinations used. Earlier studies using a Malaysian accession of *O. rufipogon *(IRGC 105491) have indicated varying frequencies of SSR polymorphism with *indica *(~60%) [13,17] and *japonica *(90%) [15] recurrent parents. The lower percentage polymorphism may be due to a higher degree of genetic similarity between *O. rufipogon *and *O. sativa *used in this study compared to those used earlier.

### Marker segregation

The expected genotypic ratio in the BC_2_ population would be 3:1 for homozygous IR 58025A : heterozygous IR 58025A/*O. rufipogon *(87.5% IR 58025A alleles to 12.5 *O. rufipogon *alleles). Out of the 80 marker loci, 28.75% (23 markers) were skewed towards one or the other parent resulting in an allele frequency of 83.26% IR 58025A alleles to 16.74% *O. rufipogon *alleles (Table 2). While 12.5% (10 markers) were skewed towards *O. sativa *parent, 16.25% (13 markers) were skewed (X_2 _> 6.6, p < 0.01) towards *O. rufipogon*.. The skewed markers were distributed on chromosomes 1, 2, 3, 5 and 8 with most of the markers on chromosome 2.

**Table 2:** Chi square values of the markers showing segregation distortion in the test cross progeny

**Marker**	**Chromosome**	**X^2^**	**Chr. Position^a^**	**Skewness^b^**
RM84	1	24.88**	0.0	IR 58025A
RM243	1	8.28*	98.1	IC 22015
RM212	1	19.47**	209.8	IR 58025A
RM14	1	11.92**	268.4	IR 58025A
RM262	2	42.54**	98.6	IC 22015
RM207	2	10.64*	227.5	IR 58025A
RM211	2	47.40**	0.0	IC 22015
RM53	2	19.47**	23.2	IC 22015
RM8	2	28.83**	42.3	IC 22015
RM240	2	24.88**	182.3	IC 22015
RM183	2	17.81**	102.4	IC 22015
RM55	3	8.28*	193.7	IC 22015
RM60	3	23.00**	0.0	IC 22015
RM7	3	17.81**	91.6	IR 58025A
RM251	3	30.95**	108.6	IC 22015
RM203	3	11.92**	173.1	IR 58025A
RM22	3	14.72**	27.5	IR 58025A
RM13	5	14.23**	24.8	IC 22015
RM169	5	28.85**	62.4	IC 22015
RM249	5	21.19**	71.1	IR 58025A
RM164	5	8.28*	87.0	IR 58025A
RM230	8	35.37**	144.2	IC 22015
RM44	8	10.64*	47.4	IR 58025A

^a^Location of the marker on the chromosome in centiMorgans

^b^Skewed marker segregation towards the *O. sativa *(IR 58025A) or *O. rufipogon *(IC 22015) parent

*Significant at p < 0.01

**Significant at p < 0.001

### QTL analysis

**Figure 2:**
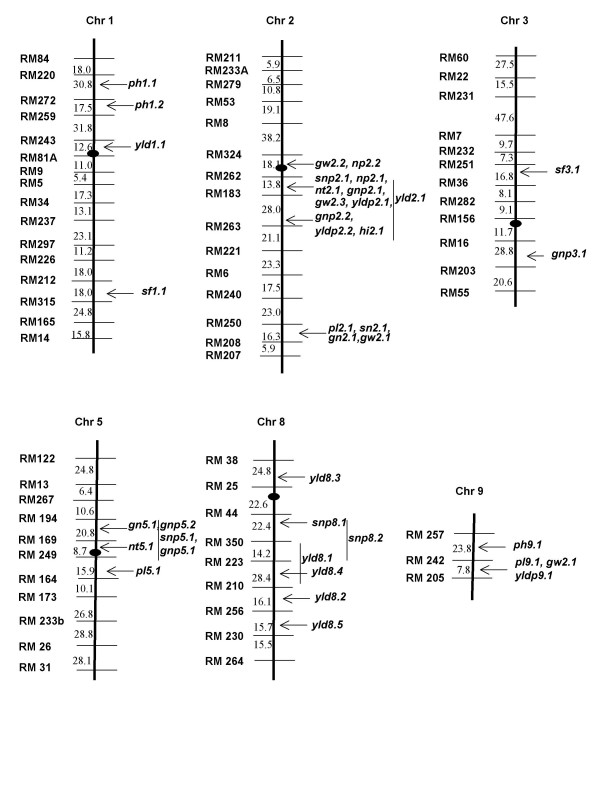
Distribution of QTLs on the molecular linkage maps constructed based on BC_2 _testcross population of IR 58025A (*O. sativa*) × IC 22015 (*O. rufipogon*) **ph**: Plant height, **nt**: Number of tillers per plant, **np**: Number of panicles per plant, **pl**: Panicle length, **sn**: Spikelet number per panicle, **snp**: spikelet number per plant, **gn**: Grain number per panicle, **gnp**: Grain number per plant, **sf**: Spikelet fertility, **gw**: Grain weight, **yldp**: Yield per plant, **hi**: Harvest index, **yld**: Plot yield.

A total of 39 QTLs were identified using composite interval mapping (CIM) and interval mapping (IM). CIM analysis detected fewer QTLs (25 QTLs) than IM (31 QTLs). While 17 QTLs (43.58%) were detected by both the methods, IM identified 14 QTLs (35.89%) exclusively and 8 QTLs (20.51%) were only detected by CIM (Table 3). Single marker analysis identified a total of 45 QTLs for the 13 traits studied [See additional file 3]. Forty two out of the 45 QTLs identified by single marker analysis were either identified by CIM or IM, so these will not be discussed separately. Three QTLs, *sf1.1, spp1.1 *and *hi1.1 *were only identified by SMA. The variation in the number of QTLs detected by different methods has been previously reported for interspecific crosses involving *O. rufipogon *[15,17]. The 39 QTLs were distributed on chromosomes 1, 2, 3, 5, 8 and 9 (Figure 2).

**Table 3:** QTLs related to yield and yield components detected in an IR58025A × *O. rufipogon *(IC 22015) population

**Trait**	**Chromosome**	**Marker Interval**		**CIM**			**IM**		
					
			**Allele effect**	**LOD**	**R^2^**	**Additive effect**	**LOD**	**R^2^**	**Additive effect**
**Plant Height**									
*ph1.1*	1	RM220 – RM272	IC 22015	5.32	17.48	-17.33	5.46	21.12	-19.09
*ph1.2*	1	RM272 – RM259	IC 22015	4.2	6.82	-2.45	3.82	5.87	-2.03
*ph9.1*	9	RM257 – RM242	IR 58025A	3.7	5.63	9.53	4.21	7.408	10.83
**Number of tillers per plant**									
*nt2.1*	2	RM262 – RM183	IC 22015				2.99	11.11	-1.28
*nt5.1*	5	RM169 – RM249	IC 22015	2.53	5.9	-1.06			
**Number of panicles per plant**									
*np2.1*	2	RM262 – RM183	IC 22015	2.5	6.8	-1.023			
*np2.2*	2	RM324 – RM262	IC 22015				3.23	10.81	-1.28
**Panicle length**									
*pl2.1*	2	RM250 – RM208	IC 22015	10.9	19.28	-9.53	10.76	20.85	-9.16
*pl5.1*	5	RM249 – RM164	IC 22015	6.61	18.93	-9.04	6.66	20.85	-9.16
*pl9.1*	9	RM242 – RM205	IC 22015	8.11	17.3	-9.24	9.48	20.85	-9.16
**Spikelet number per panicle**									
*sn2.1*	2	RM250 – RM208	IR 58025A	4.20	19.13	103.53	4.57	19.71	106.56
**Spikelet number per plant**									
*snp2.1*	2	RM262 – RM183	IC 22015				3.13	12.27	-321.31
*snp5.1*	5	RM194 – RM249	IC 22015	3.98	11.79	-413.25			
*snp5.2*	5	RM169 – RM249	IC 22015				2.52	10.29	-386.74
*snp8.1*	8	RM44 – RM350	IR 58025A	3.11	6	383.53			
*snp8.2*	8	RM44 – RM223	IR 58025A	-	-	-	2.83	6.1	381.67
**Grain number per panicle**									
*gn2.1*	2	RM250 – RM208	IR 58025A				3.32	16.65	72.52
*gn5.1*	5	RM194 – RM169	IC 22015	2.98	6.12	-5.67	3.45	7.65	-6.82
**Grain number per plant**									
*gnp2.1*	2	RM262 – RM183	IC 22015	2.79	5.42	-171.64	3.68	12.5	-256.41
*gnp2.2*	2	RM183 – RM263	IC 22015				3.31	6.5	-186.41
*gnp3.1*	3	RM16 – RM203	IR 58025A	3.14	9.8	248.31	2.71	9.98	241.4
*gnp5.1*	5	RM194 – RM249	IC 22015	5.73	12	-285.18	3.35	6.5	-193.2
**Spikelet fertility**									
*sf1.1*	1	RM212 – RM315	IR 58025A	3.26	5.2	2.45			
*sf3.1*	3	RM251 – RM36	IR 58025A	4.35	6.7	1.45	3.72	5.78	1.27
**Grain weight**									
*gw2.1*	2	RM250 – RM208	IC 22015	3.25	10.4	-5.21			
*gw2.2*	2	RM324 – RM262	IR 58025A				2.91	7.16	82.41
*gw2.3*	2	RM262 – RM183	IR 58025A				3.17	10.8	0.98
*gw9.2*	9	RM242 – RM205	IC 22015	3.21	13.95	-4.73			
**Yield per plant**									
*yldp2.1*	2	RM262 – RM183	IC 22015	4.38	12.21	-3.54	4.35	14.2	-3.79
*yldp2.2*	2	RM183 – RM263	IC 22015				3.59	7.05	-2.70
*yldp9.1*	9	RM242 – RM205	IC 22015				4.18	23.2	-13.84
**Harvest index**									
*hi2.1*	2	RM183 – RM263	IC 22015	3.12	5.83	-2.85	2.76	5.64	-2.8
**Plot yield**									
*yld1.1*	1	RM243 – RM81A	IC 22015	4.23	6.98	-3.98	3.87	5.86	-3.09
*yld2.1*	2	RM262 – RM263	IC 22015	31.92	38.46	-216.04	35.33	50.47	-238.51
*yld8.1*	8	RM350 – RM210	IC 22015	3.86	4.67	-67.45			
*yld8.2*	8	RM210 – RM256	IC 22015	3.35	3.98	-62.92	4	10.88	-103.54
*yld8.3*	8	RM38 – RM25	IC 22015				4.56	7.99	-89.13
*yld8.4*	8	RM223 – RM210	IC 22015				7.02	20.24	-138.96
*yld8.5*	8	RM256 – RM230	IC 22015				6.42	15.35	-134.53

### Interaction among QTLs

A two-way test to detect epistatic interactions between marker loci was performed using the EPISTAT software [26]. The analysis identified a total of 15 interactions consisting of 20 markers spread across 8 different chromosomes (Table 4). These markers did not contribute to the phenotype singly but had a significant effect on the phenotype in combination with another marker indicating strong G × G interactions. This may be one of the reasons for the transgressive segregants obtained.

**Table 4:** Significant two-way interactions between marker loci determined using EPISTAT program

**Trait**	**Marker 1**		**Marker 2**		**MC-test^#^**
	
	**Name**	**Chromosome**	**Name**	**Chromosome**	
NT	RM282	3	RM210	8	0.002
PL	RM243	1	RM8	2	0.001
SN	RM22	3	RM169	5	0.001
SNP	RM282	3	RM210	8	0.001
GN	RM211	2	RM263	2	0.000
GNP	RM262	2	RM169	5	0.002
	RM169	5	RM214	7	0.000
SF	RM36	3	RM340	6	0.000
HI	RM5	1	RM240	2	0.001
	RM272	1	RM211	2	0.002
	RM272	1	RM50	6	0.000
	RM60	3	RM257	9	0.005
PY	RM263	2	RM183	2	0.000
	RM263	2	RM264	8	0.077
	RM183	2	RM38	8	0.073

^#^Monte Carlo simulation using EPISTAT program to evaluate significance of interactions

## Discussion

### Marker segregation

The allele frequency in a BC_2 _population without selection would be 87.5% IR 58025A alleles to 12.5% *O. rufipogon *alleles. Twenty three markers (28.75%) were skewed towards one or the other parent resulting in an allele frequency of 83.26% IR 58025A alleles to 16.74% *O. rufipogon *alleles. Ten marker loci (12.5%) were skewed towards *O. sativa *parent, whereas, 13 markers (16.25%) had over representation of *O. rufipogon *alleles. Skewness of markers towards one of the parents has been documented for interspecific as well as intersubspecifc crosses in rice [14-19], [27-29]. A comparison of the results with earlier studies involving *O. rufipogon *revealed that the percentage of skewed markers was lower compared to that reported by Moncada *et al *[15] (37.6%) and Thompson *et al *[18] (42.5%) and higher compared to Septiningsih *et al *(21.4%) [17]. All the three previous studies used same accession of *O. rufipogon *(IRGC 105491)) but different recurrent parents. This suggests that the polymorphism percentage is relative and depends on parental combination. Skewness towards the elite parent could have been due to the intensity of selection imposed in the BC1 generation, while, skewness towards *O. rufipogon *may be due to reduced recombination and linkage drag in some regions of an interspecific population [30,31]. While, segregation distortion of RM251 and RM7 on chromosome 3 may be due to their proximity to the gamete abortive gene, *ga3 *[32], the deviation from the mendelian ratio of RM249 and RM44 might be due to the presence of these markers close to the centromeres of chromosome 5 and 8 respectively. Some of the chromosomal regions may have been distorted due to the selection imposed in BC1. The segregation distortion, towards IR 58025A, of marker loci RM84 on chromosome 1, RM207 on chromosome 2 and RM22 on chromosome 3, which exist in close proximity to dwarfing genes *d18, d29 *and *d50 *respectively [32] may be due to the selection for semi-dwarf plant height in BC1 generation. The distortion of RM13, RM169 and RM262 (markers associated with QTLs for number of tillers) towards *O. rufipogon*, may be due to the selection imposed on BC1 for high tiller number, a trait that was superior in *O. rufipogon*. RM13 was also shown to exhibit segregation distortion towards *O. rufipogon *in an earlier study [17].

### Trait correlations

The present study confirms that major components follow significant positive relationship with yield. Most of the trait correlations confirm with those reported earlier for studies involving *O. rufipogon*. Grain weight was negatively associated with both spikelet number per panicle and grain number per panicle [13,17,18]. In the present study, the correlation between grain weight and yield was non significant as also reported earlier for an IR64/*O. rufipogon *cross [17], However other studies on *O. rufipogon *report a positive correlation between yield and grain weight [13,18]. There is no correlation detected in the present study between panicle number and yield, however, a positive correlation between these two traits was reported in an IR64/*O. rufipogon *derived cross [17].

### *O. rufipogon *derived QTLs for yield improvement

*Oryza rufipogon *alleles had a beneficial effect on 74% of the QTLs obtained for yield and yield components in the present study. This is a higher percentage than documented for interspecific crosses in rice. In previous studies involving *O. rufipogon*, alleles from wild species had beneficial effect in 35–58% of the QTLs [13,15,17,18]. The higher percentage reported here might indicate the presence of a larger number of favorable alleles in this accession of *O. rufipogon *compared to the one used in the previous studies. Alternatively, IR 58025A might have inferior alleles at many of the loci compared to the *O. rufipogon *alleles or the alleles introgressed from the wild species may interact better with the IR 58025A background compared to the *O. sativa *accessions used earlier. The intensive selection in the BC1 for higher tiller number, a superior trait in *O. rufipogon*, may be another reason for the increased contribution of wild alleles.

The *O. rufipogon *alleles contributed to an increase in panicle length (*pl2.1, pl5.1, pl9.1*), number of tillers (*nt2.1, nt5.1*), number of panicles (*np2.1, np2.2*), spikelet number per plant (*snp2.1, snp5.1, snp5.2*), grain number per panicle (*gn5.1*), grain number per plant (*gnp2.1, gnp2.2, gnp5.1*), grain weight (*gw2.1, gw9.1*), yield per plant (*yldp2.1, yldp2.2, yldp9.1*), harvest index (*hi2.1*) and plot yield (*yld1.1, yld2.1, yld8.1, yld8.2, yld8.3, yld8.4, yld8.5*). The *O. rufipogon *alleles also resulted in an increase in plant height (*ph1.1, ph1.2*), however, they did not enhance spikelet fertility and spikelet number per panicle. It is interesting because, the population had transgressive segregants for both the traits, in spite of the inferiority of *O. rufipogon *for these traits. The alleles in IR 58025A may be dominant at this loci compared to the alleles from *O. rufipogon*. However, despite its inferiority for the trait, the alleles from the wild species had beneficial effect on grain weight indicating that the alleles contributing to grain weight might interact positively with the genetic background of IR 58025A.

### Interaction among QTLs

An analysis to identify the potential epistatic interactions between marker loci, using EPISTAT software [26], identified 20 markers resulting in 15 two-way interactions (Table 4). All these markers had no effect on the trait singly but resulted in an enhanced effect when combined with another marker. The resulting G × G interactions between these markers may be one of the reasons for the appearance of transgressive segregants in the population. Several chromosomal regions were associated with more than one trait, indicating linkage or pleiotropic effects. For example, the QTLs *gnp2.2 *and *yldp2.2*, associated with an increase in grain number per plant and yield per plant respectively were located in the same region on chromosome 2. Similarly, the region associated with *nt2.1 *which controlled an increase in number of tillers was linked to *np2.1, gnp2.1, yldp2.1, hi2.1 *and *yld2.1 *controlling an increase in number of panicles, grain number per plant, yield per plant, harvest index and plot yield respectively. The *O. rufipogon *alleles had beneficial effect on all these traits. However, the same region is associated with a negative QTL from *O. rufipogon*, *gw2.3*, resulting in decreased seed weight. At a different chromosomal region, *O. rufipogon *allele associated with a QTL *gw2.1*, leading to an increase in grain weight is linked to two negative QTLs, *sn2.1 *and *gn2.1*, which result in decrease in spikelet number per panicle and grain number per panicle. The reverse is true for the region associated with another QTL for grain weight, *gw2.2*. This negative QTL from *O. rufipogon *is linked with two QTLs corresponding to grain number per plant, *gnp2.1 *and yield per plant, *yldp2.1*, where the *O. rufipogon *alleles had positive effect. It is very interesting that the same chromosomal region associated with a positive QTL for grain weight coincides with negative QTLs for spikelet number and grain number and vice versa. As grain weight is negatively correlated with spikelet number and grain number, it is tempting to speculate that the same QTL might contribute to both the phenotypes. Further characterization of this region by fine mapping and identification of genes underlying it will throw more light on whether the same set of genes, regulated differentially, or an entirely different set of genes govern these phenotypes. The association of positive and negative QTLs to the same chromosomal regions was earlier reported for studies involving *O. rufipogon *where the positive traits for grain weight and panicle length together and panicle length alone were linked with negative QTLs for plant height and broken rice respectively [17,19]. *In lieu *with the association of the positive and negative QTLs to same chromosomal regions, a careful selection will be needed to avoid negative characteristics in the crop improvement process.

### Comparison with QTLs from other wild species

A comparison of the QTLs obtained with the other wild species including *O. rufipogon, O. glaberrima *and *O. glumaepatula *revealed that 27 out of 39 QTLs obtained in this study had congruent occurrences with QTLs reported earlier (Table 5). The QTLs that overlap with other studies fall into two categories: i) QTLs that share similar map position and mapped to same trait and ii) QTLs that share a similar map position, but mapped to a different trait. The QTL for panicle length, *pl2.1*, is mapped to the same region under same name in a study involving *O. rufipogon*/Jefferson [18], while it is associated with yield components like grains/panicle and yield in a cross involving V20A/*O. rufipogon *[13]. However, the *O. rufipogon *alleles contributed to positive effect in both the cases. In case of *pl9.1*, QTLs under same name and associated with same chromosomal regions were reported previously in three separate studies involving *O. rufipogon *(IRGC 105491) [13,17,18]. However, the same region is associated with a negative QTL for panicle length (pnl) in a study involving another accession (P 16) of *O. rufipogon *[14]. This indicates an accession based variation in the alleles at this locus, with alleles derived from accessions IRGC 105491 and IC 22015 superior to the alleles from *O. rufipogon *accession, P 16. The alleles from *O. rufipogon *increased yield at *yld1.1, yld2.1, yldp2.1, yldp2.2 *and *yld8.1*. While, QTLs with same names as *yld1.1 *and *yld8.1 *were reported in similar regions in a cross V20A/*O. rufipogon*, the beneficial effect of *yld2.1, yldp2.1 *and *yldp2.2 *coincided with an increase in panicle length [13]. The position of *yld8.1 *overlaps with another yield component, grains per panicle, *gpp8.1*, in a cross involving Jefferson/*O. rufipogon *[18]. The QTLs for grain weight, *gw2.1 *and *gw9.1 *shared same names and had orthologous regions with the QTLs identified in V20A/*O. rufipogon *cross [13]. While, *O. rufipogon *had beneficial effect on grain weight in the present study, they had a negative effect in the earlier study, suggesting that the alleles at this locus might be superior to those reported earlier. However, a negative QTL from grain weight in this study, *gw2.3*, is associated with panicle length, *pl2.1*, in case of [13] where the *O. rufipogon *alleles had beneficial effect.

**Table 5:** Comparison of QTLs with other studies involving wild rice species

**Chr./marker**	**QTLs in this study**	**QTLs identified in other wild species**						**Ref**
**Chromosome 1**								
RM220 – RM272	*ph1.1*	*spp1*	*gyp1*	*fgp1*	*gypa1*			16
		*sp1*	*al1*					14
RM272 – RM259	*ph1.2*	*dth1.1*						18
RM243 – RM81A	*yld1.1*	*yld1.1*						13
		*BR*						19
RM212 – RM315	*sf1.1*	*BR*						19
		*ph1.1*	*pl1.1*					17
		*pss1.1*	*pth1.2*	*ph1.2*	*pl1.1*			18
								
**Chromosome 2**								
RM250 – RM208	*pl2.1,sn2.1,gn2.1,gw2.1*	*yld2.1*	*gpp2.1*					17
		*gl2.1*	*gw2.1*					18
		*ph2.1*	*gpl2.1*					15
		*ppl2.1*	*gpl2.1*	*yld2.1*				13
RM262 – RM183	*nt2.1,np2.1,yld2.1,gw2.3*	*dtf2*						16
*gnp2.1,yldp2.1, snp2.1*	*pl2.1*							13
RM262 – RM263	*gnp2.1,yldp2.1,hi2.2*	*dtf2*	*spp2*	*fgp2*				16
		*pl2.1*						13
		*al2*						14
		*HR*	*CR*	*BR*				19
								
**Chromosome 3**								
RM36 – RM251	*sf3.1*	*CR*						19
		*dth3.2*	*sh3.2*					18
		*kl3.1*						20
		*amy3*						21
								
**Chromosome 5**								
RM194 – RM249	*gn2.1,snp5.1,gnp5.1*	*gw5.1*						13
**Chromosome 8**								
RM44 – RM350	*snp8.1*	*tnr8*	*dnr8*	*plh8*				16
		*sh8.1*						18
		*cp8.1*						20
								
RM350 – RM210	*yld8.1*	*GD*						19
		*gpp8.1*	*ph8.1*					18
		*ph8.1*	*pl8.1*	*gpl8.1*	*yld8.1*			13
								
**Chromosome 9**								
RM257 – RM242	*ph9.1*	*gw9.1*						13
RM242 – RM205	*pl9.1,gw9.1*	*pnl*						14
		*pl9.1*	*spp9.1*					13
		*dyg*						19
		*pl9.1*						17
		*gw9.1*	*gpp9.1*	*spp9.1*	*pl9.1*	*yld9.1*	*tt9.1*	18

### Comparison of QTLs across Oryza species

The present study identified a total of 39 QTLs. Thirty QTLs have corresponding occurrences with QTLs reported earlier, while, 9 QTLs (*nt2.1, nt5.1, snp5.1, hi2.1, yldp2.1, yldp9.1, yld2.1, yld8.1, yld8.5*) are novel and reported for the first time. The results are on the expected lines, as new parental combinations especially involving exotic species are likely to unfold novel variability. Of the three QTLs detected for plant height, the *O. rufipogon *alleles increased plant height at two loci while another QTL decreased plant height. All the three QTLs have been reported previously (Table 6). The similarity of the regions associated with QTLs for plant height with other studies involving *O. rufipogon, indica *and *japonica *cultivars indicates that the location of alleles for plant height are conserved across different genetic and environmental backgrounds. The two QTLs for tiller number, *nt2.1 *and *nt5.1*, identified in this study are novel and have no correspondences with QTLs reported earlier for this trait. This indicates that these may be a potentially new set of alleles specific for this accession of *O. rufipogon*. All the QTLs for number of panicles have been reported earlier (Table 6).

**Table 6:** Comparison of QTLs across *Oryza *species

**QTL**	**QTLs reported in earlier studies**
*ph1.1*	PTHT [33,34], *ph1 *[35], *qPH-1 *[36], *ph1-1 *[37]
*ph1.2*	PTHT [33,34,36], *ph1 *[35], *ph1-1 *(10), *ph1 *[38]
*ph9.1*	*ph9 *[37,38]
*np2.1*	*tns2 *[35]
*np2.2*	PNNB [35,40,57]
*pl2.1*	PNL [53], pl2.1 [18]
*pl5.1*	PNL [40], QPI5 [53]
*pl9.1*	*qPI9b *[53], *pl9.1 *[13], *pl9.1 *[17], *pl9.1 *[18]
*sn2.1*	SPKNB [53]
*snp2.1*	*tns5 *[35]
*snp5.1*	SPKNB [34,40,53]
*snp8.1*	SPKNB [40], *tns5 *[35]
*snp8.2*	SPKNB [40], *tns5 *[35]
*gn2.1*	*gpp2.1 *[17]
*gn5.1*	FGRNB [41]
*gnp2.1*	FGNRB [39, 55] *nfg *[35]
*gnp2.2*	*fgp2 *[16]
*gnp3.1*	*gp3 *[41]
*gnp5.1*	*qNFGP-5-1 *[56]
*sf1.1*	SF [51], *pss1.1 *[18]
*sf3.1*	SF [51], *S3b *[52], SF [11]
*gw2.1*	GW [39], *gw2.1 *[41]
*gw2.2*	GW [39], *tgwt *[35], GW [54]
*gw2.3*	*kw2-2 *[55]
*gw9.1*	*gw9.1 *[18]
*yldp2.2*	GRYLDPPL [35,39,40]
*yld1.1*	GRYLD [39], *yld1.1 *[13]
*yld8.1*	GRYLD [39], *yd8 *[41], *yld8.1 *[13]
*yld8.3*	GRYLD [40]
*yld8.4*	GRYLD [35,39], *yd8 *[41]

All the three QTLs for panicle length, *pl2.1, pl5.1 *and *pl9.1*, were trait enhancing and overlapped with the regions identified earlier for the same trait (Table 6). This is in agreement with earlier studies where *O. rufipogon *alleles had a positive effect [13,16-18]. The large number of studies implicating a similar region as *pl9.1 *indicate that this region has a similar predictable effect on the phenotype irrespective of the genetic background. Two QTLs were identified for grain number per panicle (*gn2.1, gn5.1*) and one for spikelet number per panicle (*sn2.1*) in the present study. The alleles from *O. rufipogon *had a negative effect on the QTLs on chromosome 2, whereas, they enhanced the number of grains per panicle on chromosome 5. A similar negative effect of *O. rufipogon *alleles had been reported for grain number per panicle, on chromosome 2, in a cross involving IR64/*O. rufipogon *[17]. The negative effect across *O. rufipogon *accessions indicates the superiority of the *O. sativa *alleles at this locus.

Five QTLs were identified for spikelet number per plant and 4 QTLs for grain number per plant. All the four QTLs for grain number per plant were reported earlier, while only four of the five QTLs for spikelet number per plant are documented (Table 6). The *O. rufipogon *alleles had negative effect on *snp8.1, snp8.2 *and *gnp3.1 *whereas, they had beneficial effect on all the other QTLs. The QTL for grain number per plant, *gnp2.2*, coincided with a QTL reported for the same trait (fgp) in a cross involving *O. glumaepatula *[16]. While, the *O. rufipogon *alleles had a beneficial effect in the present study, the *O. glumaepatula *alleles had a negative effect, indicating that this accession of *O. rufipogon *has a novel set of alleles at this locus that are superior to *O. glumaepatula*. The QTL *snp5.2 *is novel and is reported for the first time. Again, this indicates the possibility of the presence of novel alleles in this accession of *O. rufipogon*. Two QTLs, *sf1.1 *and *sf3.1*, both conferring negative effect, were identified for spikelet fertility. Both the QTLs have been reported earlier and they also are in agreement with earlier study indicating the negative effect of the *O. rufipogon *alleles on this trait [18]. All the four QTLs for grain weight have been reported earlier indicating that the allele set may be common across most of the genetic backgrounds. The *O. rufipogon *alleles contribute to positive effect for two of these QTLs (*gw2.1, gw9.1*), while the other two derive negative effect from the wild alleles. The beneficial effect at *gw2.1 *and *gw9.1 *is in contrast to what has been previously reported for this trait in a Jefferson/*O. rufipogon *cross, where the *O. rufipogon *alleles have a deprecating effect on both these QTLs [18]. This indicates that alleles at these loci may be superior in this accession of *O. rufipogon *or the same set of alleles might perform better in the IR 58025A background compared to the Jefferson background or the G × E interactions might be at play.

Six of the eight QTLs identified for yield have been reported earlier [35,39-41] suggesting that the QTLs for yield are conserved across different genetic backgrounds. Two QTLs, *yld2*.1 and *yld8.5 *are reported for the first time. The *O. rufipogon *alleles had beneficial effect on all the eight QTLs. Of the two QTLs identified for yield per plant, the *O. rufipogon *alleles were responsible for increase in yield in both the cases. While, *yldp2.1 *is novel and reported for the first time, *yldp2.2 *coincides with the regions reported earlier for this trait (Table 6). The QTL for harvest index, *hi2.1*, is novel and is reported for the first time in this study.

## Conclusion

The study while confirming the view that the progenitor species constitute the largest source of still unfolded variability for traits of complex inheritance like yield and its components has helped identify additional novel variability for yield improvement. The novel QTLs identified are good candidates for fine mapping and positional cloning studies, while, the QTLs that are mapped to regions consistent with other studies can be useful for marker-assisted transfer of these QTLs. The availability of the complete rice genome sequence and rapid advances being made in the area of genomics will help dissect and characterize yield related QTLs further. Considering the potential of yield influencing new QTLs, more research is warranted to unearth and use more and more novel yield related gene blocks hidden in closely related wild/weedy species and primitive cultivars, if the rice dependent world is to truly attain and sustain food security.

## Methods

### Choice of parents

IR 58025A, a commercial cms line developed by Directorate of Rice Research (DRR), India,  was used as a recurrent parent. IR 58025A grows to a height of 80 cm and is characterized by having long grain type and early maturity along with good milling and eating qualities. The *O. rufipogon *accession, IC22015, collected from Kerala, India, and maintained at DRR was used as a donor parent.

### Development of mapping population

An advanced backcross strategy as described in [13] was followed to develop the mapping population. A single plant of *O. rufipogon *(IC 22015) was used as a male parent and crossed to IR 58025A to generate F_1 _plants. Fourteen F_1 _plants, whose hybrid nature was confirmed with microsatellite markers were backcrossed to IR 58025B (an isogenic line of IR 58025A) used as male to produce BC_1_. Fifty BC_1 _plants, looking morphologically like IR 58025A were backcrossed to IR 58025B to produce BC_2_. Out of a population of 3000 BC_2 _plants obtained, 251 male sterile plants were randomly selected and testcrossed to KMR3, the restorer of IR 58025A to produce 251 testcross families. The 251 BC_2 _testcross families constituted the mapping population. Simultaneously, under similar conditions, a cross was made between IR 58025A and KMR3 to obtain the hybrid, KRH2, to be used as the check.

### Phenotypic evaluation of mapping population

The 251 testcross families, two parents and checks *viz*., KRH2, Jaya and IR64 were grown under irrigated conditions at DRR in an augmented block design in two replications with checks repeated after every 10 families. Each of the testcrosses and the check consisted of 40 plants planted in 4 rows of 10 plants each adopting a uniform spacing of 20 cm × 20 cm. Six plants in the middle of each of these families were evaluated for the following yield related traits: *Plant height (PH) *– length of the tallest tiller (cm) from soil surface to the tip of the panicle. *Tiller number per plant (NT) *– Total number of tillers per plant. *Panicles per plant (NP) *– Panicles with seed set exceeding 15%. *Panicle length (PL) *– length (cm) from neck to tip of the panicle. *Spikelet number per panicle (SN) *– number of spikelets including empty and filled ones averaged over five randomly chosen panicles in each plant. *Spikelet number per plant (SNP) *– total number of spikelets including empty and filled ones in each plant computed as average number of spikelets per panicle × number of productive tillers. *Grain number per panicle (GN) *– number of filled spikelets per panicle averaged over five randomly chosen panicles in each plant. *Grain number per plant (GNP) *– number of filled spikelets in a plant computed as average number of filled spikelets per panicle × number of productive tillers per plant. *Spikelet fertility (SF) *– ratio of filled spikelets to the total number of filled and unfilled spikelets per panicle, expressed in percentage. *Grain weight (GW) – w*eight (g) of 1000 filled spikelets, averaged over six samples taken from the bulk-harvested grain from each plant. *Harvest index (HI) *– ratio of filled grains to biomass (filled grains, unfilled grains and straw of the plant) in terms of weight (g) expressed in percentage. *Grain yield per plant (Yldp) *– weight (g) of filled grains per plant. *Grain yield (Yld) *– weight (g/kg) of filled grains harvested from each testcross family (40 plants) extrapolated to tonnes per hectare.

### Trait correlations

Correlations between character pairs were computed at p < 0.05 and p < 0.01 in Excel using trait averages.

### DNA extraction

DNA was extracted from two months old leaf tissue using the protocol of Dellaporta [42].

### Parental polymorphism and linkage map construction

A set of 210 randomly selected microsatellite markers (Donated by Rockefeller Foundation to EAS) spanning all the 12 chromosomes were screened among the *O. sativa *and *O. rufipogon *parents. A total of 80 polymorphic microsatellite markers separated by an average distance of 15.37 cM were used to analyze the 251 testcross progeny. Linkage maps were constructed using the Mapmaker version 3.3 [43] following Kosambi Function [44]. Linkage groups were determined using 'group' command with an LOD score of 3.0 and a recombination fraction of 0.5. Order of the markers for each group was determined using 'order' and 'ripple' commands. Assignment of linkage groups to the respective chromosomes was done based on the rice maps developed at Cornell University [18,45].

### QTL analysis

QTLs were analyzed using single marker analysis (SMA), interval mapping (IM) and composite interval mapping (CIM). Single marker analysis wasperformed by regression of field performance on marker genotypes using standard analysis of variance (ANOVA) procedure at a statistical threshold of p < 0.01 and assuming regular segregation of wild and cultivated alleles in the testcross families. The proportion of observed phenotypic variance attributable to a particular QTL was estimated as the difference between the mean of the segregants having the *O. rufipogon *allele and the mean of the segregants that did not have the *O. rufipogon *allele. The phenotypic variance over the check KRH2 was also calculated in a similar manner. QTL analysis by interval mapping (IM) and Composite interval mapping (CIM) [46] was done using QTL Cartographer 3.0 [47]. The significant threshold value for identification of a QTL (both for IM and CIM) was determined based on permutation tests at a significance level p <0.01 [48]. Based on 1000 permutations for each trait, the threshold for IM and CIM corresponded to minimum LOD score value of 2.5. The proportion of phenotypic variance (R2) and additive effect were determined for each trait. The deviations from the expected mendelian ratio was calculated using MapDisto software [49] and the digenic interactions between marker loci were determined using EPISTAT software [26]. The QTL nomenclature followed was as reported in [50].

### Note

The material used in this study can be obtained from Prof E.A. Siddiq, Honorary Professor, Center for DNA Fingerprinting and Diagnostics, Nacharam, Hyderabad, 500 076, India. The raw data used for analysis can be obtained from Dr. M. Pradeep Reddy, Department of Biology, McMaster University, Hamilton, ON, Canada.

E-mail: reddyp@mcmaster.ca

## Authors' contributions

**MPR **participated in the design, carried out the field studies and marker studies, analyzed the data and darafted the manuscript. **NS **participated in the coordination of the study and helped in the design of the study, analysis of data and drafting the manuscript. **VLNR **carried out the field studies, marker studies and helped in analysis of data. **EAS **conceived of the study and participated in its design and coordination. All authors read and approved the final manuscript.

## Supplementary Material

Additional File 1 Transgressive Segregants Click here for file

Additional File 2 Correlation Data Click here for file

Additional File 3 Single Marker QTLs Click here for file
