# Supplementary material for: Identification and mapping of yield and yield related QTLs from an Indian accession of Oryza rufipogon
Source: BMC Genet. 2005 Jun 13;6:33. doi: 10.1186/1471-2156-6-33 (PMC1181812; doi:10.1186/1471-2156-6-33)
Supplement: Additional File 1 — Transgressive Segregants [file 1471-2156-6-33-S1.ppt]

## Slide 1
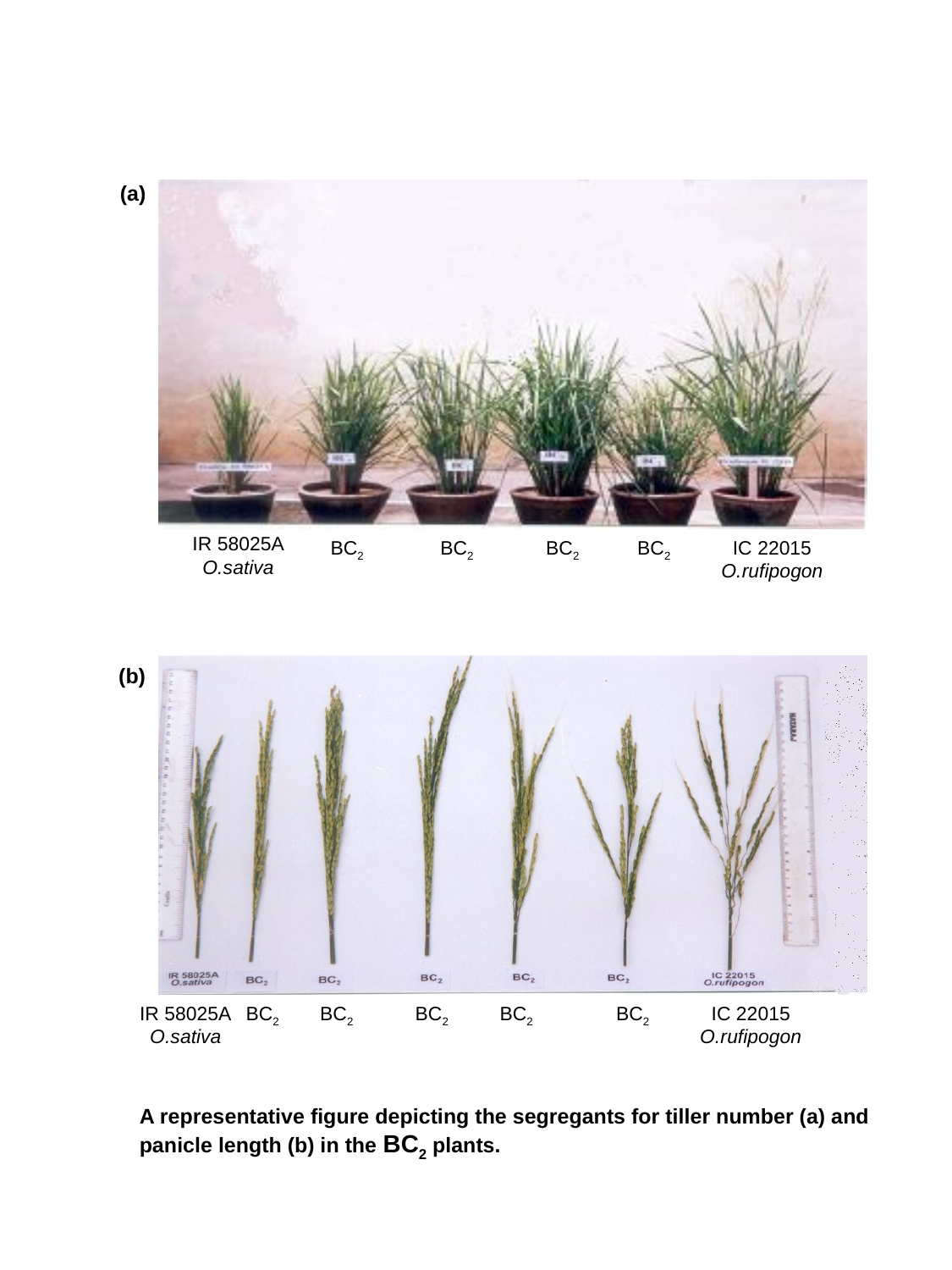

(a)
IR 58025A
O.sativa
BC2
BC2
BC2
BC2
IC 22015
O.rufipogon
(b)
IR 58025A
O.sativa
BC2
BC2
BC2
BC2
BC2
IC 22015
O.rufipogon
A representative figure depicting the segregants for tiller number (a) and panicle length (b) in the BC2 plants.
